# Utilizing post-stack seismic inversion for delineation of gas-bearing sand in a pleistocene reservoir, baltim gas field, nile delta, Egypt

**DOI:** 10.1038/s41598-024-78186-9

**Published:** 2024-11-28

**Authors:** Ali S. EL-SAYED, Walid M. MABROUK, Ahmed M. METWALLY

**Affiliations:** Geophysics Department, Faculty of Science, Cairo University, Giza, 12613 Egypt

**Keywords:** Post-stack seismic inversion, El Wastani formation, Gas-bearing sand, Baltim Field, Offshore Nile Delta, Geology, Geophysics

## Abstract

Given useful rock properties, gas sands generally appear as bright events on seismic data. Unfortunately, partially saturated gas sands tend to be of similar brightness to fully saturated gas sands so cannot be distinguished easily. Seismic inversion can sometimes help, given adequate seismic data and a robust understanding of rock physics. This study employs both post- and pre-stack seismic inversion techniques to estimate gas in place (OGIP) and evaluate reservoir properties within the Pleistocene gas sandstone reservoir of the Baltim Field, focusing specifically on the Kanaria prospect. The primary objectives are to estimate gas volumes, assess reservoir properties, and identify optimal well locations. Post-stack inversion is used to detect changes in acoustic impedance, which is essential for hydrocarbon identification, while pre-stack inversion provides insights into rock properties such as density. This approach helps to differentiate true gas sands from other geological features, like water layers or lithological anomalies, which can be misinterpreted as gas zones due to similar seismic responses or fluid variations. Although seismic indicators such as root mean square (RMS) amplitude and AVO analysis can suggest potential gas zones, they may also be affected by similar lithological effects, complicating accurate interpretation. The study confirms the presence of gas-bearing sands in the El Wastani and estimates an OGIP of approximately 0.4 Gm³ for well WB-1 and 6.6 Gm³ for well TERSA-1 ST. By providing detailed reservoir characterization and improving gas estimation accuracy, the findings support informed decision-making for drilling locations and enhance the potential for successful gas production in the Baltim Field.

## Introduction

The hydrocarbon potential of the Nile Delta Basin was first recognized in the 1960s. The Baltim Field, spanning 500 km² in the offshore area, is situated on the northern extension of the El-Qar’a main channel, known as the Abu Madi paleovalley^1,2^. Positioned between 31.02° and 31.44°E longitude and 31.62° and 31.94°N latitude, the Baltim Field is approximately 500 km from the Egyptian shoreline.

Geologically, the Baltim Field is characterized by the El Wastani Formation, deposited during the Plio-Pleistocene cycle, transitioning between the Mit Ghamr and Kafr El Sheikh formations^3^. Structural mapping of the El Wastani Formation reveals a northeast-trending complex basin influenced by fault systems oriented northeast, north-northeast, and northwest, with a well-developed Neogene hinge zone in the mid-Delta region. Thickness varies from 200 to 400 m^4,5^.

**Fig. 1:**
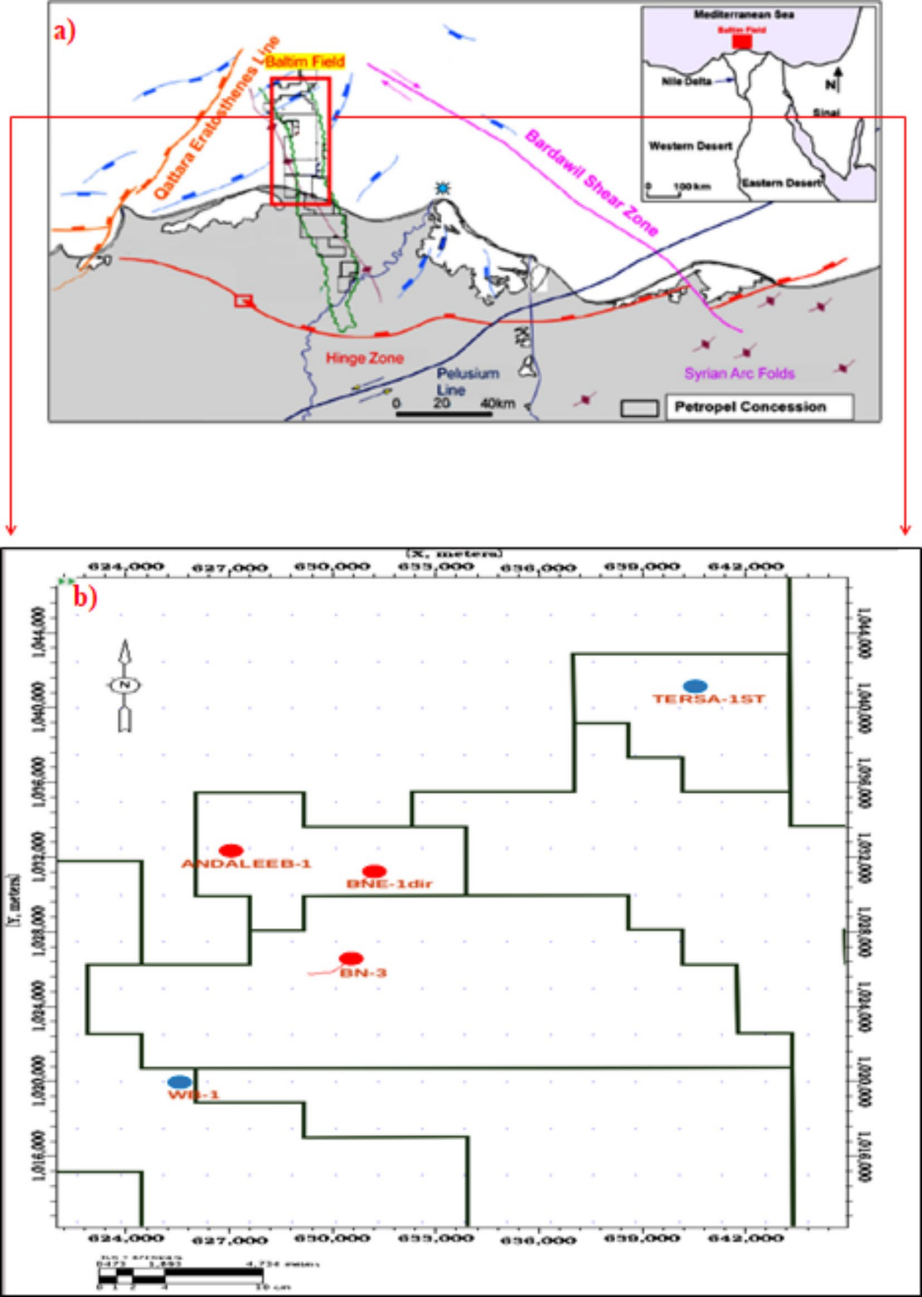
(**a**) Location map of the central Nile Delta, Egypt. (**b**) A base map showing the well locations, created by the Landmark Workstation, Decision Space Desktop (DSD).

Production at the Baltim Field began with significant discoveries, including Baltim North in 1995 and Baltim East in 1993 (Fig. 1a). Initial production commenced in April 2000, aligning with the development of Baltim North and Baltim East. Well BN1, crucial for interconnecting these fields, started full-scale production in November 2005^6^.

Developed by the International Egyptian Oil Company (IEOC), the Baltim Field achieved an initial production rate of 150 MCF/d by late 1999 and aimed for a plateau capacity of 460 MCF/d. It is one of five recently developed Mediterranean fields contributing to a combined plateau capacity of 1,500 MCF/d. Amoco’s discovery of the Baltim Field in July 1993 marked a significant find in the Eastern Mediterranean^7^. Over the past five decades, the Nile Delta Basin has been prolific for gas discoveries across geological epochs, including the Oligocene, Pliocene, and Pleistocene^8–16^.

The offshore Nile Delta has emerged as a critical area for gas production, leveraging high-quality three-dimensional seismic data^17,18^, along with insights from successful deep-water explorations and appraisal wells^19,20^. These efforts provide a comprehensive understanding of sedimentation and erosion stages in upper Pliocene deep-marine slope channels, with significant volumes of gas condensate stored in the Baltim Eastern and North gas reservoirs^21^.

The Baltim Field is covered by several seismic surveys. In 2005, they were combined to generate four field-specific angle stack volumes^6^. Seismic inversion techniques play a pivotal role in enhancing resolution^22^, interpreting lateral velocity variations, and developing reservoir models^23^. Post-stack data analysis methods, such as sparse-spike, model-based, and colored inversion techniques^24–27^, provide crucial insights into acoustic impedance (AI) and lithology cubes, which are essential for identifying hydrocarbon traps and understanding reservoir morphologies. These methods help locate gas sands by identifying areas with the lowest acoustic impedance. Additionally, pre-stack seismic inversion calculates density cubes, enabling precise identification of areas with the lowest density, which are often indicative of optimal well locations for targeting reservoirs such as the Pleistocene Kanaria^17,28,29^. These findings are further validated against well log data parameters^29^.

The primary goal of this study is to optimize well placement for the Pleistocene Kanaria anomaly in the Baltim Field. Class III AVO behavior is observed at the reservoir level in amplitude maps from seismic sub-stacks, indicating the presence of gas in the Pleistocene Kanaria anomaly. However, observations made on standard attribute maps do not fully mitigate the risk of partial saturation gas or poor hydraulic conductivity (Kh). To address these uncertainties, the study uses both post- and pre-stack inversion techniques to predict Vp, Vs, and density. These predictions are integrated with AVO analysis and cross-plotting to minimize exploration risk for the Kanaria prospect^25–27^.

Furthermore, the study explores prospective development opportunities in the El Wastani sandstone reservoirs at the Baltim Field, highlighting its potential for future gas exploration and production^17,29,30^.

## Geological setting

**Fig. 2:**
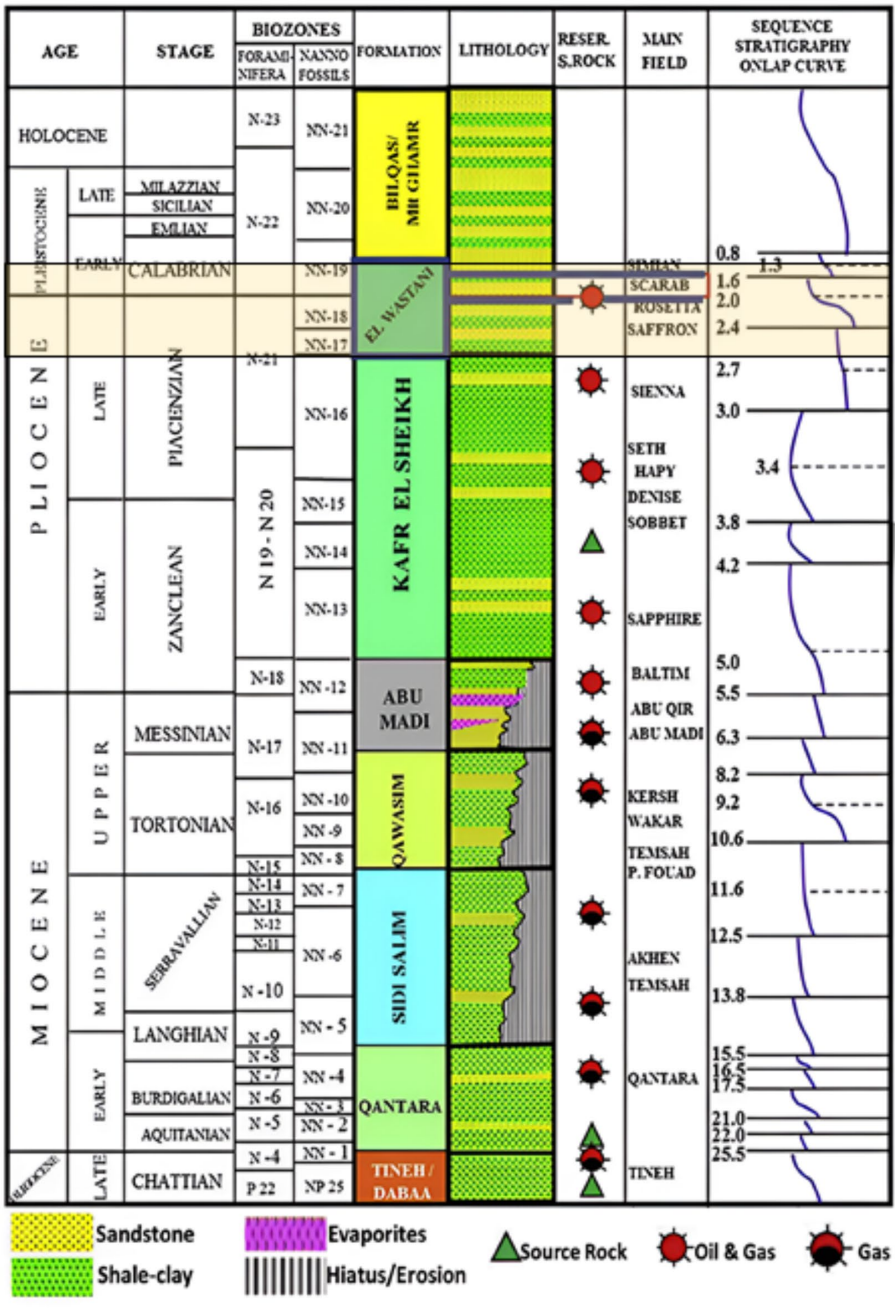
Nile Delta stratigraphic column and hydrocarbon system, modified by^31^.

The stratigraphic sequence of the Baltim field in the Neogene section comprises a siliciclastic succession that includes the Tineh, Qantara, Sidi Salim, Qawasim, Abu Madi, Kafr El Sheikh, El Wastani, Mit Ghamr, and Bilqas Formations^31^ (Fig. 2). This study focuses primarily on the El Wastani Formation within the Pliocene-Pleistocene cycle identified by Rizzini^3^, which is further divided into the Kafr El Sheikh and El Wastani Formations^9,18,32,33^.

Sedimentation in the Nile Delta during the Pliocene era included a deep marine series of Early Pliocene age overlying the “fluvio-marine” Abu Madi and Qawasim Formations in the Central sub-basin and the Rosetta “evaporites” in the Eastern sub-basin. The marine deposits predominantly consist of shaly deposits that are part of the Kafr El Sheikh Formation. Sedimentation progresses upwards within the formation, transitioning into coastal and fluvial sands characteristic of the El Wastani Formation, marking both the upper Pliocene and lower Pleistocene cycles. The Pliocene cycle is overlain by the Pleistocene/Holocene transgression cycle, represented by the Mit Ghamr and Bilqas Formations^34^.

The stratigraphic nomenclature used for these units follows the revisions outlined in^35^, arranged in chronological order from oldest to youngest.

### Kafr El Sheikh formation (pliocene)

This formation consists of deep marine continental slope shales interspersed with turbidite sands. The well Kafr El Sheikh-1, which ranges from 1277 to 2735 m^35^, represents the type section.

### *El Wastani Formation (early Pleistocene to late Pliocene*)

The El Wastani Formation at the El Wastani-1 well is approximately 123 m thick, extending from 1009 to 1132 m. It primarily comprises thick quartzose sands with interbedded argillaceous layers^33,35^. The formation’s depositional environment is on the continental shelf, which is of particular interest in this research.

## Methodology

The study employed advanced seismic interpretation techniques utilizing 3D seismic volumes organized into four-angle stacked volumes: full angle, near (0°–15°), mid (15°–30°), and far (30°–45°) angles. The term “3D seismic volumes” refers to the organized arrangement of seismic data in three dimensions, which can include various orientations such as inlines and crosslines.

The primary goal of this study is to optimize well placement for the Pleistocene Kanaria anomaly at the Baltim Field. This was achieved through various seismic volume stacks and amplitude attributes, complemented by Amplitude versus Offset (AVO) cross-plot methods. To enhance accuracy and reduce uncertainties associated with post-stack inversion, deterministic wavelets were employed in pre-stack inversion methods to compute P-impedance (Zp) and S-impedance (Zs)^36,37^. The integration of these seismic volumes with data from two wells was performed using the Landmark Workstation, Decision Space Desktop (DSD).

Pre-stack inversion provided detailed estimates of reservoir properties, while post-stack seismic inversion techniques, including model-based (sparse-spike) and colored inversions, were utilized to refine the seismic volumes and confirm the existence of gas by identifying low acoustic impedance values. Acoustic impedance analysis provided initial insights into gas sands by facilitating the detection of lateral lithological variations and the interpretation of reflective boundaries, which were further refined by pre-stack inversion methods^38,39^. The seismic inversion procedure involved transforming migrated seismic volumes into acoustic impedance volumes, integrating both seismic and well data along with structural and stratigraphic features^37,40,41^. The incorporation of high-frequency well data and seismic velocities enabled the extraction of ultra-low and low-frequency components, facilitating a more detailed analysis and interpretation.

### Data preparation

**Fig. 3:**
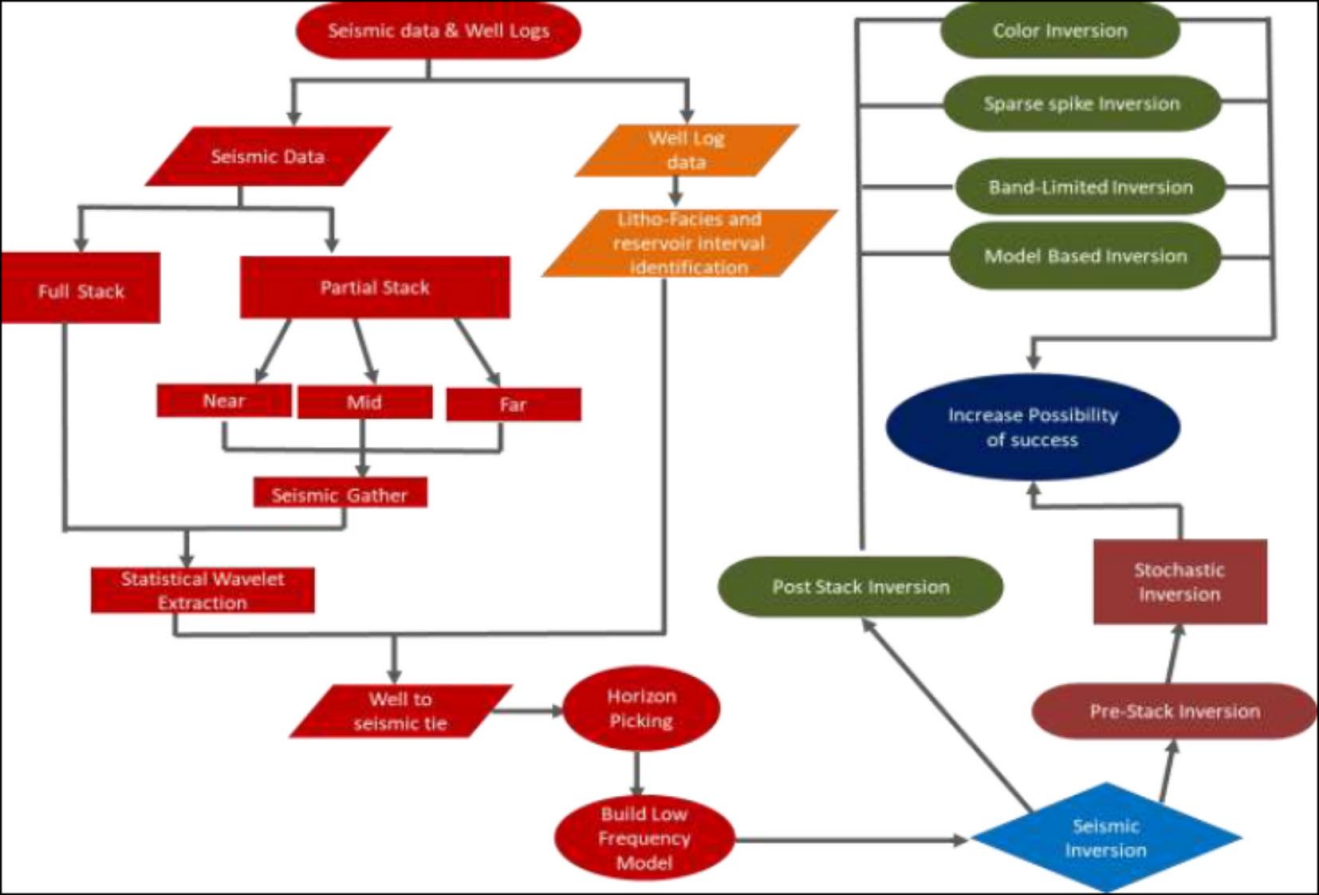
Research Workflow: From Seismic Data to Success Assessment. This figure shows the process from acquiring seismic data and well logs, through inversion techniques, to evaluating success probability and identifying optimal drilling locations.

This study follows a structured workflow that is typical for conventional seismic processing (Fig. 3):

#### Seismic data processing

Seismic data preparation began with the processing of high-quality seismic volumes obtained from the four-angle stacked datasets. The seismic data underwent Pre-Stack Time Migration (PSTM) to enhance image resolution and reduce travel-time distortions. Additionally, Surface-Related Multiple Elimination (SRME) was applied to reduce noise and eliminate multiple reflections, preparing the data for subsequent analysis.

#### Well log conditioning and correction

Well logs from two wells within the study area were conditioned to ensure accurate impedance modeling. This conditioning involved corrections for fluid effects and alignment with seismic data. Key parameters such as shear velocities (Vs) and bulk density were integrated to provide reliable inputs for inversion.

##### Applying fluid substitution

We simulated various conditions through fluid substitution to develop an optimal Rock Physics model. This step was crucial for predicting how different pore fluids affect seismic velocities and impedance, which is essential for distinguishing partial gas saturation and improving the accuracy of gas reservoir identification. It also helped in optimizing the location of discovery wells.

**Fig. 4:**
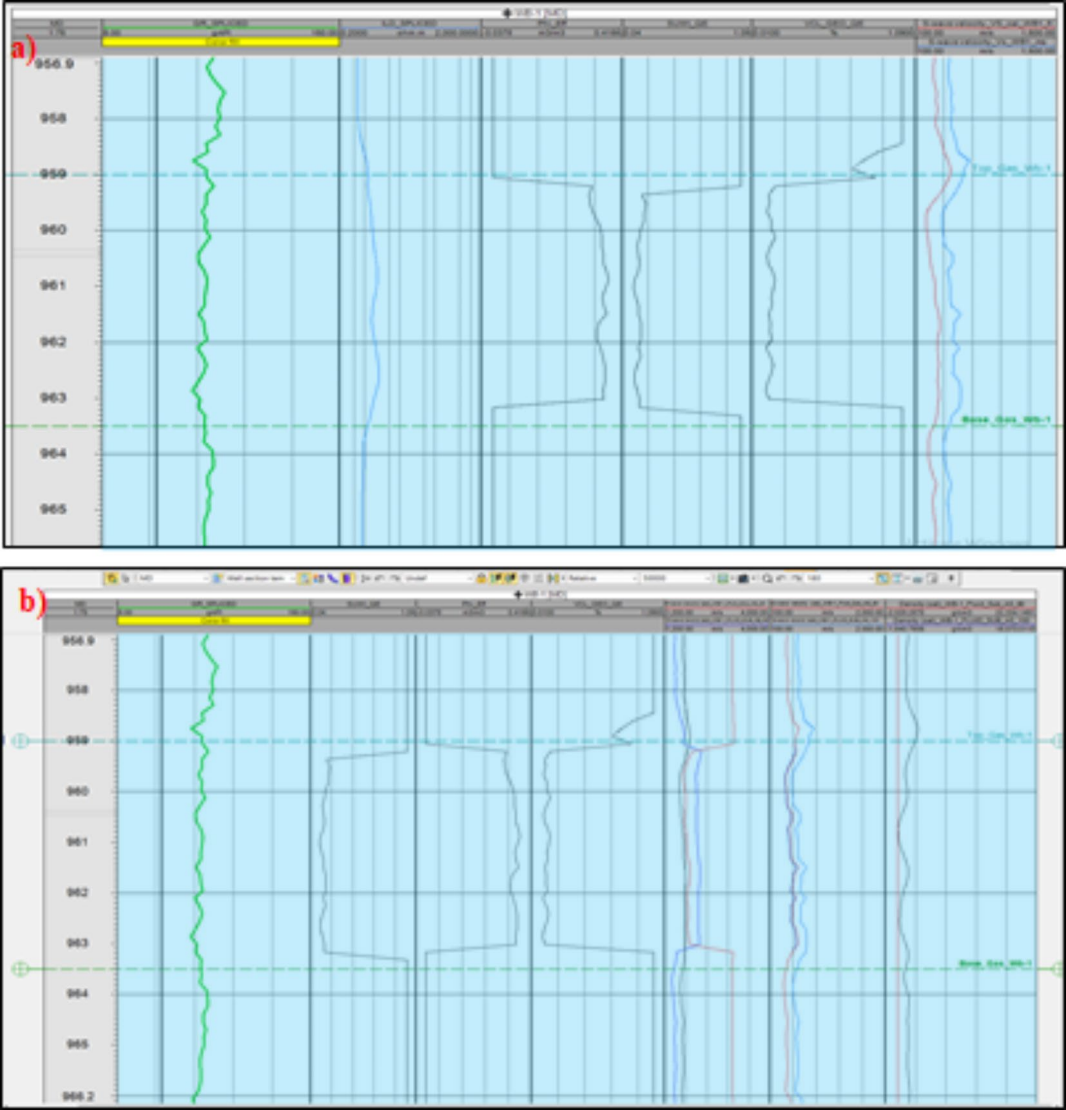
(**a**) Shear Velocity (Vs) calculation for the WB-1 well (El Wastani Formation), showing the relationship between actual and calculated velocities. (**b**) Fluid Substitution results for WB-1, demonstrating how pore fluids impact seismic velocities and impedance, aiding in the development of the low-frequency model. The black curves represent in-situ conditions, the red curves show 60% gas substitution, and the blue curves indicate 100% gas substitution, applied using Petrel software.

Fluid substitution is a critical process used to evaluate how different pore fluids impact seismic velocities and impedance. Guided by Gassmann’s equations^42^, we simulated the effects of varying fluids, such as gas and water, on seismic responses. For instance, in wells like WB-1, we estimated shear wave velocity (Vs) and compared it with the actual Vs measurements, as illustrated in Fig. 4a. We conducted fluid substitution scenarios to examine the differences between gas and water saturation (Sw = 100% and Sw = 60%), which allowed us to analyze the effects on seismic velocities and rock moduli. In cases where direct density measurements were not available, we calculated density from porosity logs using mass balance equations. This methodology ensured a comprehensive understanding of how fluid variations influence seismic data, thereby enhancing the inversion process for the El Wastani prospect (Fig. 4b).

#### Synthetic seismogram and extracting wavelets

**Fig. 5:**
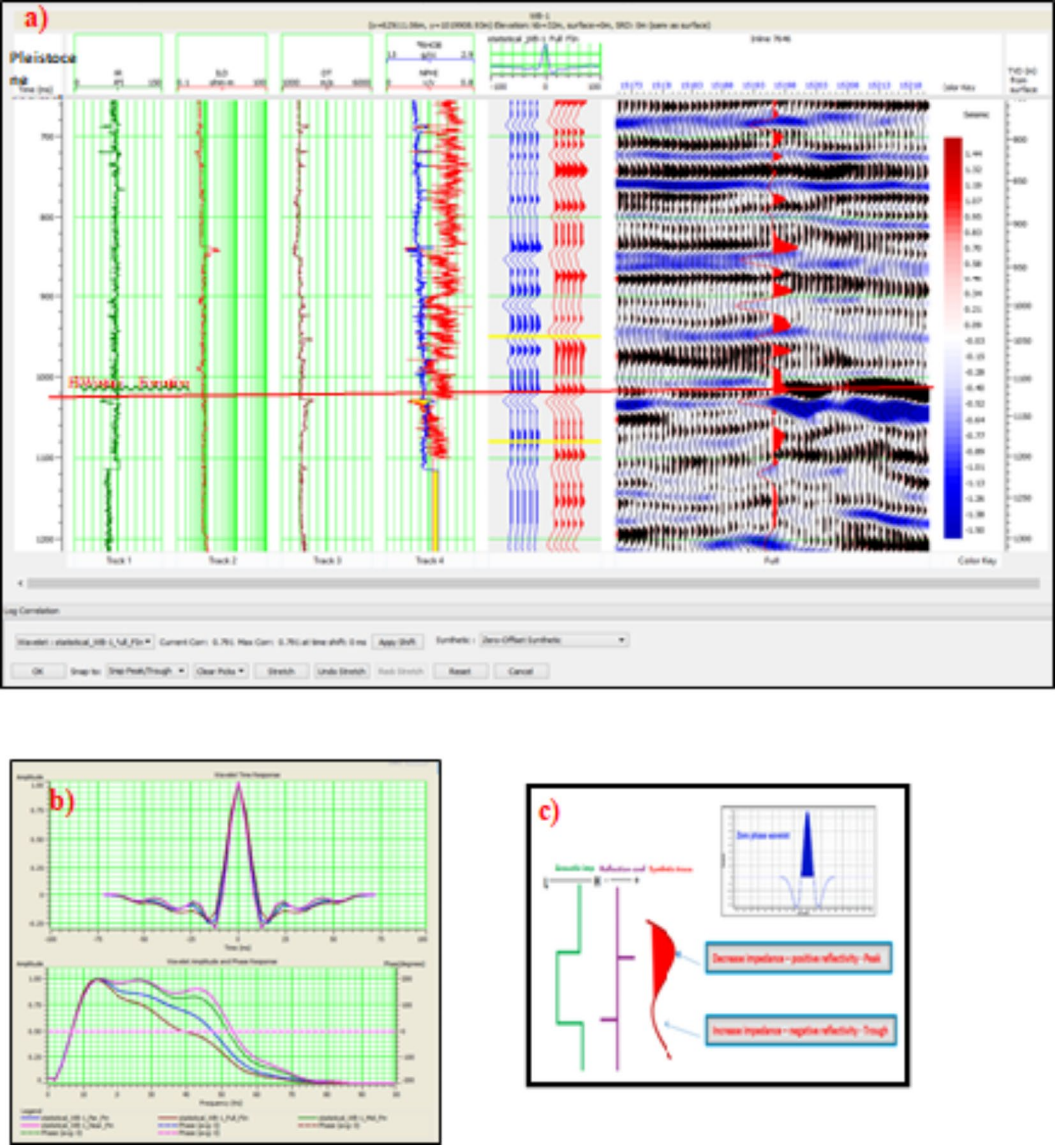
(**a**). Seismic tie for WB-1 well, performed using Hampson-Russell Software, extracted from the full seismic domain. (**b**) Combined statistical wavelets from the WB-1 well, displaying near (violet), mid (green), far (blue), and full offset (brown) seismic volumes. (**c**) Phase and polarity of WB-1 well, from Hampson-Russell AVO Training Manual, 2008.

This section focuses on depth conversion and the extraction of RMS amplitudes from a 10-meter window just below the top of the gas sand reservoir in the Pleistocene El Wastani Formation. Key geological surfaces were interpreted from the processed seismic data, and amplitude attributes were extracted to assist in identifying subsurface features. A refined depth-dependent velocity model, replacing the initial constant velocity, was used for seismic depth conversion, improving accuracy by reflecting variations in seismic velocities. The resulting depth contour map for the gas sand reservoir ranges from 810 to 850 m, enhancing the precision of the depth interpretation and distinguishing the Pleistocene anomaly from surrounding formations (Fig. 5). The high anomaly, despite its elevated values, is not located at the highest structural point. This anomaly is considered primarily trapped by a stratigraphic trap, specifically a stratigraphic pinchout, as the anomaly in the El Wastani Formation is laterally surrounded by shale layers. This observation is supported by seismic sections passing through the anomaly, which demonstrate juxtaposition with shale, creating a barrier that encapsulates the gas reservoir. Several geological factors may contribute to the lack of gas migration updip. First, the presence of lateral shale layers surrounding the El Wastani anomaly acts as a significant barrier, preventing buoyancy-driven migration of gas toward higher structural positions. Additionally, stratigraphic pinchouts within the formation may create localized traps that retain gas, further inhibiting upward movement. The interplay between these sealing features and the stratigraphy of the reservoir plays a critical role in maintaining the gas within its current location. Future analysis of these barriers will provide further insights into the dynamics of gas migration in this area.

**Fig. 6:**
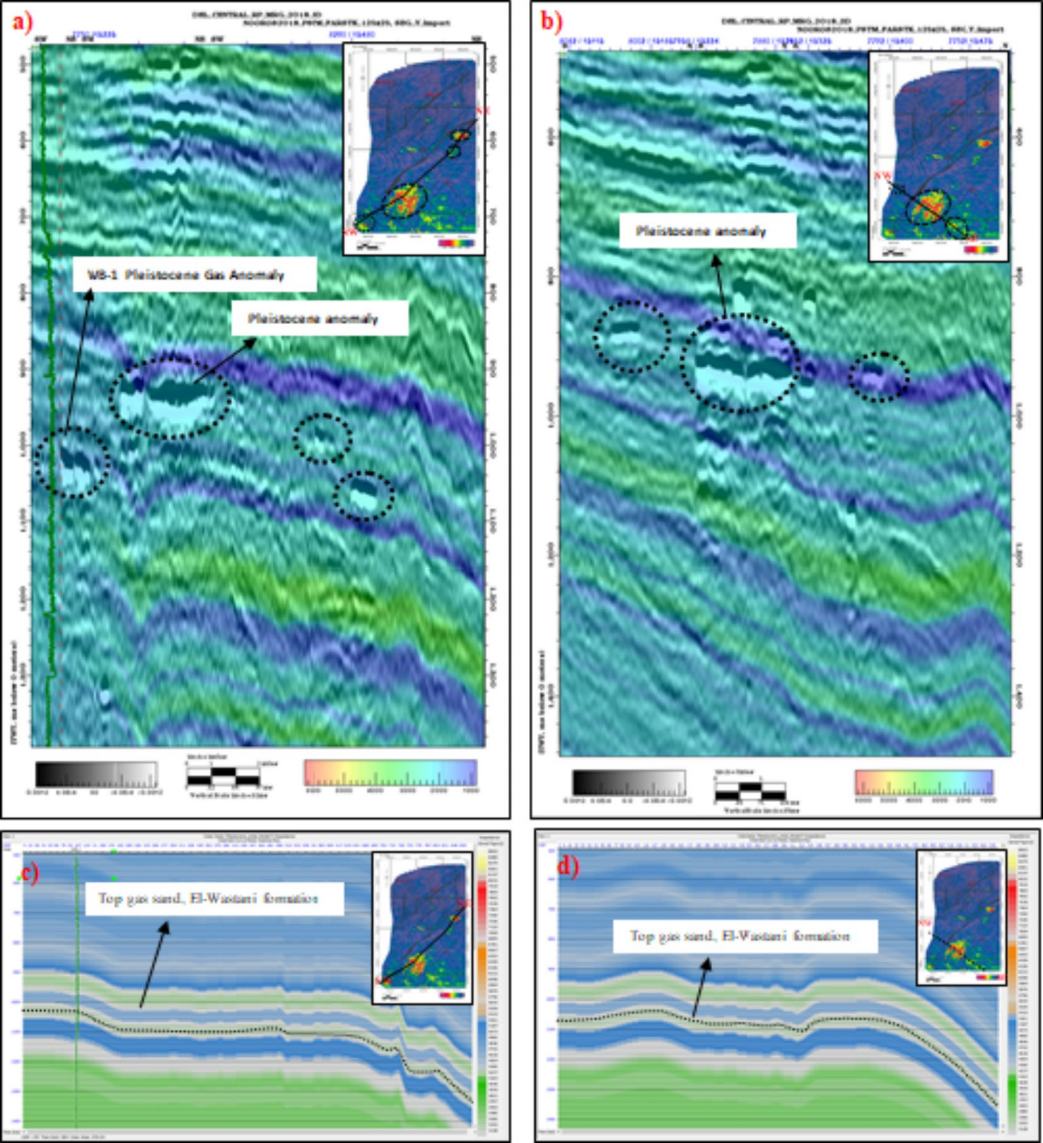
(**a**) SW-NE arbitrary seismic section with the velocity model derived from 3D stacking velocities (frequency range: 10–50 Hz). (**b**) NW-SE arbitrary seismic section with the same velocity model. (**c**) Low-frequency model for P-impedance from Post-Stack Inversion compared to actual impedance at well WB-1, with a regional far amplitude/depth map (0-10 ms) for the Pleistocene anomaly (frequency range: 0–10 Hz). (**d**) Another view of the low-frequency model for P-impedance with the same depth map and frequency range (0–10 Hz).

Seismic data was correlated with well logs to validate and calibrate the seismic signals. Given the limited depth coverage of the extracted wavelets, additional wavelets were also extracted from various angle stacks to ensure a robust analysis and minimize uncertainties. Figure 6a and b present seismic sections across the prospect, each overlaid with a velocity model derived from seismic data, which operates within a frequency range of 10–60 Hz. Figure 6c and d show the same seismic sections compared with a low-frequency model obtained from well logs, operating within a frequency range of 0–10 Hz. This comparison illustrates the distinction between the high-frequency velocity model and the low-frequency model. The velocity model offers detailed insights from seismic data, while the low-frequency model provides a broader view based on well log data. This approach helps differentiate between the two models and enhances interpretation accuracy for the Kanaria prospect by integrating both high-resolution seismic and broad-spectrum well log information.

**Fig. 7:**
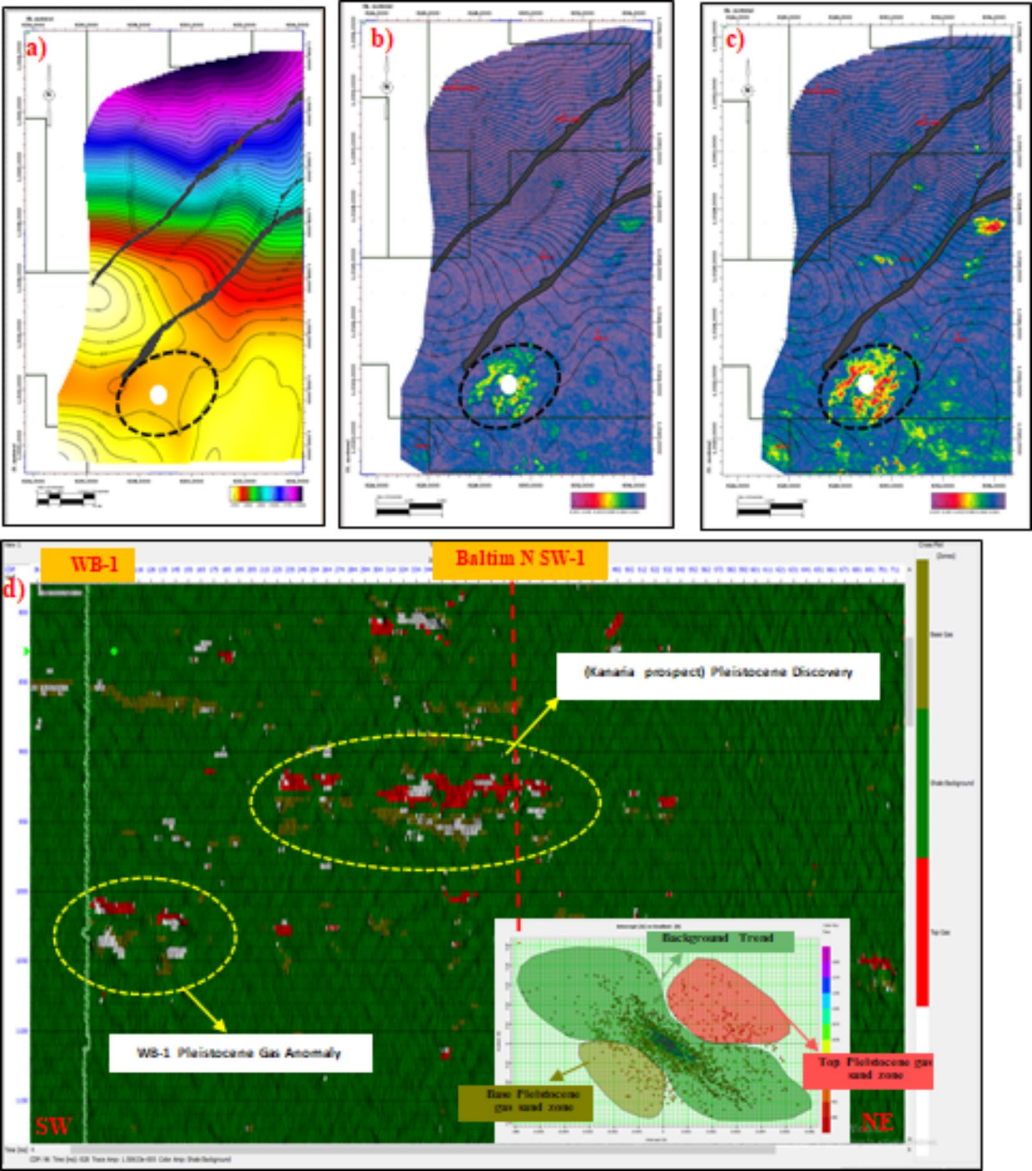
(**a**). Pleistocene Anomaly, Structure Depth Map (contour interval = 10 m). (**b**) Pleistocene Anomaly, RMS amplitude (10 ms interval) below the top reservoir, near amplitude/Depth Map. (**c**) Pleistocene Anomaly, RMS amplitude (10 ms interval) below the top reservoir, far amplitude/Depth Map. (**d**) Color-coded section with cross-plot for Pleistocene Anomaly in El Wastani Fm. (Kanaria prospect) and WB-1 Pleistocene Gas Anomaly, highlighting gas zones.

RMS amplitude extraction showed brighter amplitudes in the far-stack compared to the near-stack, consistent with Class III AVO behavior observed in well data AVO crossplots. The adjustment of the amplitude extraction window improved the accuracy of the analysis, providing clearer Class III AVO results (Figs. 6c and 7b). The application of the AVO crossplot has been clarified and simplified. An A/B crossplot derived from well data at WB-1 illustrates the relationship between intercept and gradient, helping to identify zones corresponding to the top and base of the gas sand. On the seismic section, the top of the gas sand is highlighted in red, while the base is colored in light grey. Water-bearing sands and shale are depicted in green (Fig. 7d). Further validation is provided by VSh (volume of shale) and Sw (water saturation) logs from WB-1, ensuring the accuracy of the AVO analysis and the identification of gas sand intervals.

#### Surface interpretation and amplitude extraction

This section focuses on depth conversion and the extraction of RMS amplitudes from a 10-meter window just below the top of the gas sand reservoir in the Pleistocene El Wastani Formation. Key geological surfaces were interpreted from the processed seismic data, and amplitude attributes were extracted to assist in identifying subsurface features. Figure 6a and b present seismic sections across the prospect, each overlaid with a velocity model derived from seismic data, which operates within a frequency range of 10–60 Hz. Figure 6c and d show the same seismic sections compared with a low-frequency model obtained from well logs, operating within a frequency range of 0–10 Hz. This comparison illustrates the distinction between the high-frequency velocity model and the low-frequency model. The velocity model offers detailed insights from seismic data, while the low-frequency model provides a broader view based on well log data. This approach helps differentiate between the two models and enhances interpretation accuracy for the Kanaria prospect by integrating both high-resolution seismic and broad-spectrum well log information.

A refined depth-dependent velocity model, replacing the initial constant velocity, was used for seismic depth conversion, improving accuracy by reflecting variations in seismic velocities. The resulting depth contour map for the gas sand reservoir ranges from 810 to 850 m, enhancing the precision of the depth interpretation and distinguishing the Pleistocene anomaly from surrounding formations (Fig. 7a).

RMS amplitude extraction showed brighter amplitudes in the far-stack compared to the near-stack, consistent with Class III AVO behavior observed in well data AVO crossplots. The adjustment of the amplitude extraction window improved the accuracy of the analysis, providing clearer Class III AVO results (Figs. 6c and 7b). The application of the AVO crossplot has been clarified and simplified. An A/B crossplot derived from well data at WB-1 illustrates the relationship between intercept and gradient, helping to identify zones corresponding to the top and base of the gas sand. On the seismic section, the top of the gas sand is highlighted in red, while the base is colored in light grey. Water-bearing sands and shale are depicted in green (Fig. 7d). Further validation is provided by VSh (volume of shale) and Sw (water saturation) logs from WB-1, ensuring the accuracy of the AVO analysis and the identification of gas sand intervals.

#### Depth conversion

Seismic data was initially processed in the time domain using migrated volumes. Following this processing, a velocity model derived from well logs and seismic velocities was used to perform depth conversion. This conversion allowed for precise mapping of the subsurface structure and target horizon, enhancing the accuracy of seismic interpretation.

### Petrophysical analysis

One important step in estimating hydrocarbon reserves is petrophysical evaluation. Well log analysis is crucial for assessing reservoir petrophysical parameters, including hydrocarbon saturation, water content, shale content, and porosity^21,30,43–46^.

The distinctive crossover observed in resistivity logs indicates the presence of hydrocarbons within the reservoir. Identifying the intersection of the density-porosity and neutron-porosity logs provides the most accurate method for detecting porous zones^29^.

Petrophysical parameters were determined including shale volume (VSh), total porosity (ΦT), effective porosity (ΦE), and water saturation (Sw). These parameters were analyzed to identify gas-bearing zones. VSh values were derived from gamma-ray logs, while ΦT and ΦE were estimated from neutron-density logs^47^. A modified version of the Archie equation, specifically adapted for calculating Sw in the presence of shale^48^, was used to estimate water saturation.

The modified Archie equation includes an additional conductivity parameter to account for the effect of clay on resistivity. Specifically, the dual water saturation prediction model incorporates a cementation factor (m) of 1.69 and a saturation exponent (n) of 2.0. This approach addresses the tortuosity of clay particles, which varies depending on their size and composition, and enhances the accuracy of water saturation estimates.

**Fig. 8:**
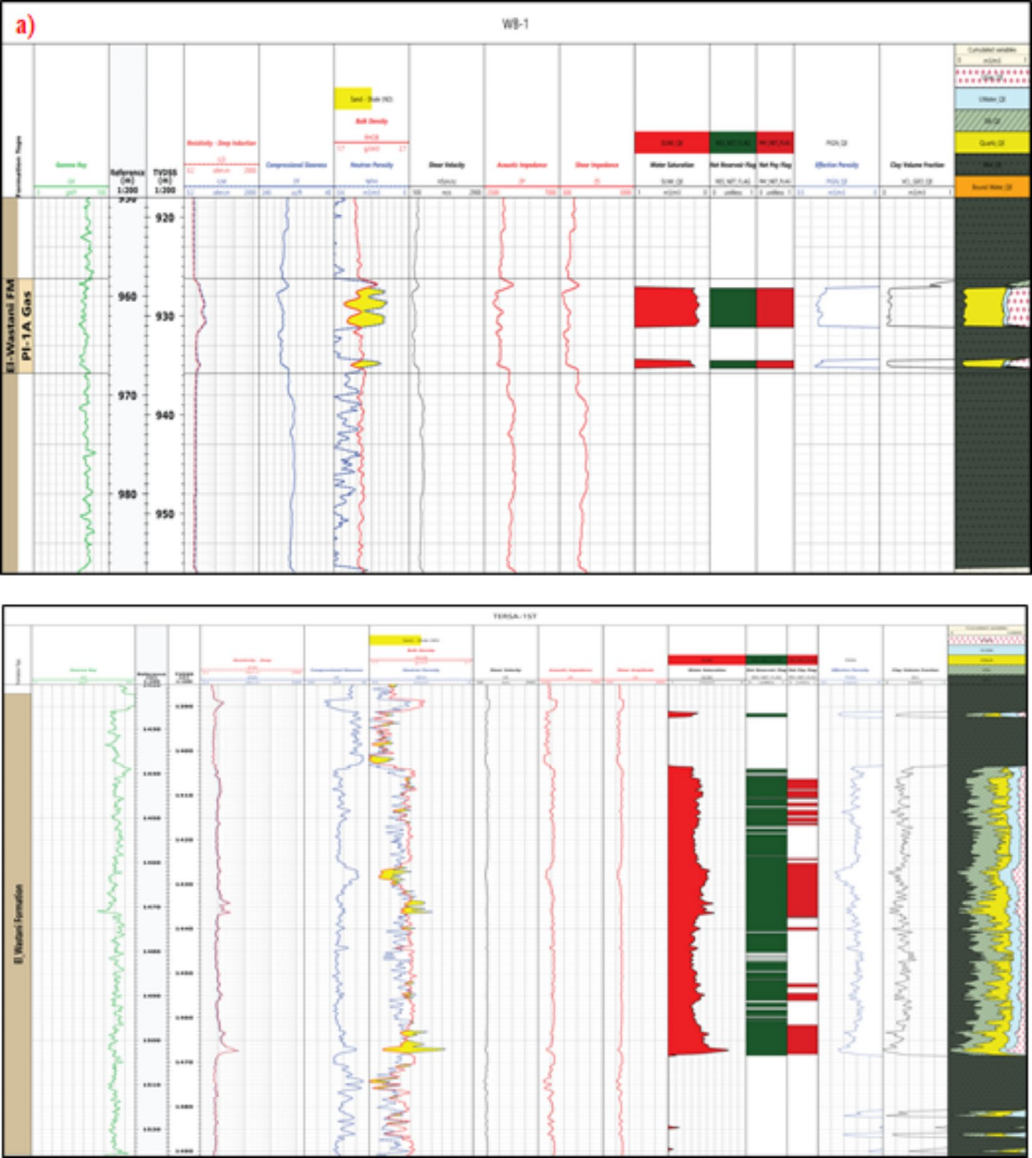
Petrophysical interpretation of WEST BALTIM-1 well (PL-1 A) (El WASTANI Formation, by applying Schlumberger’s Techlog software). (**b**) Petrophysical interpretation of Tersa-1_ST well (El WASTANI Formation, by applying Schlumberger’s Techlog software).

The El Wastani Formation is characterized by alternating sandstone and shale layers. With cutoff parameters of 18% water saturation, 32% porosity, and 40% shale volume, the WB-1 well yields approximately 4 m of net pay with 18% water saturation and 32% effective porosity. Similarly, the El Wastani Formation in the Tersa-1 ST well contains around 30 m of net pay with 54% water saturation and 25% effective porosity, based on the same cutoff criteria (Fig. 8). The petrophysical characteristics of both wells are compiled in Table 1.

**Table 1:** Summarized petrophysical parameters for WEST BALTIM-1 (WB-1) and TERSA-1_ST Wells.

Parameters	WEST BALTIM (WB-1)	TERSA-1_ST
	TVDss. (m)	MD. (m) TVDss. (m)
Intervals	959-969	1422-1505
Gross Thickness	10	83
Net Pay	4	30
Net / Growth	0.4	0.37
Por. Range (Pay)	(30-36) Av. 33 %	(20-36) Av. 25 %
SW. Range (Pay)	(18-25) Av. 18 %	(21-60) Av. 54 %

### Seismic inversion

Seismic inversion techniques, when combined with well-logged data, allow us to create detailed models of rock and fluid properties. Even without well information, seismic data can provide insights into lithology and fluid parameters^38,49^. In this study, our goal was to validate the El Wastani gas anomaly and confirm the presence of gas in the Pleistocene Kanaria anomaly at the Baltim Field using both post- and pre-stack seismic inversions.

To achieve this, we utilized an initial model to iteratively refine our geological model by comparing it with seismic data. Unlike some post-stack inversion methods, such as band-limited inversion, which do not rely on an initial model, our approach involved using models of lithology, acoustic impedance (AI), and density to identify hydrocarbon traps.

Table 2, provided separately, summarizes the post-stack and pre-stack inversion methods used in this study, including their inputs and outputs. This table offers a clear overview of the techniques applied and their associated parameters, which helps in understanding the methodology employed.

**Table 2:** Summary of post-stack and pre-stack seismic inversion methods: inputs and outputs.

Inversion Method	Type	Inputs	Outputs	Key Characteristics
Post-Stack	Colour	Seismic Data, Well Logs, Velocity Model	Impedance Models (full bandwidth), Rock Properties, Seismic Attributes	Focuses on specific frequency bands.
	Band-Limited	Seismic Data, Well Logs, Velocity Model	Impedance Models (bandlimited), Rock Properties, Seismic Attributes	Outputs are bandlimited.
	Model-Based	Seismic Data, Initial Model, Well Logs, Velocity Model	Impedance Models (full bandwidth), Rock Properties, Seismic Attributes	Uses a model to constrain inversion.
	Sparse Spike	Seismic Data, Initial Model, Well Logs	Impedance Models (full bandwidth), Rock Properties, Seismic Attributes	Resolves fine details by applying sparsity.
Pre-Stack	Simultaneous Inversion	Seismic Data (from multiple offsets), Initial Model, Velocity Model, Well Logs	Elastic Properties (full bandwidth), AVO Attributes, Volume of Shale	Provides detailed subsurface property estimates.

#### Steps for seismic inversion

##### Calculating shear velocity (vs)

For wells where shear wave velocity (Vs) was not directly measured, we estimated it from P-wave velocity (Vp) using the Greenberg-Castagna equation. Although Vs data was not directly recorded for the WB-1 well, this estimation supported the inversion process.

Creating an Initial Model: To build an accurate initial model, we generated a low-frequency component using well-log data within the seismic time frame of 1800 to 2300 milliseconds. This low-frequency model, created by applying a low-pass filter (0 to 10 Hz) to high-frequency data, was vital for capturing broad subsurface features with long wavelengths. This model incorporated frequency components absent in the seismic data^3,50,51^. While seismic data (10–60 Hz) provided high-frequency information, it lacked the low-frequency impedance trend (0–10 Hz) necessary for this analysis. To address this, well-log data from two gas exploration wells—WEST BALTIM-1 (WB-1) and TERSA-1_ST were used to establish the low-frequency impedance trend. These wells included density and sonic logs, which were critical for constructing a high-frequency model that was then interpolated and extrapolated within the seismic data.

The accuracy of the low-frequency model depended heavily on the interpolation process and the variability of the well data. If wells exhibit similar characteristics, interpolation between them is straightforward. However, for this case study, significant variations in the two wells used as input presented challenges in defining where changes occurred and whether they should be gradual or abrupt, introducing uncertainty into the low-frequency model and potentially impacting the inverted volumes.

To address these challenges, seismic horizons relevant to the Pleistocene gas sand horizon were carefully selected for the inversion process. The low-frequency impedance model was generated by applying a low-pass filter (0 to 10 Hz) to the high-frequency model, constructed using the interpolated acoustic impedance data from the wells. This filtered low-frequency model served as the basis for the inversion, closely resembling the actual impedance.

In summary, while integrating well-log data was essential for determining the low-frequency impedance trend, the interpolation process and variability among wells were critical factors influencing the accuracy of the initial model. These aspects were considered to ensure that the low-frequency impedance model effectively supported the inversion results. To build an accurate initial model, we generated a low-frequency component using well-log data within the seismic time frame of 1800 to 2300 milliseconds. This low-frequency model, created by applying a low-pass filter (0 to 10 Hz) to high-frequency data, was vital for capturing broad subsurface features with long wavelengths.

##### Executing inversions

We performed both post- and pre-stack inversions using the initial model. By integrating these methods, we aimed to provide a consistent and reliable approach to confirming the presence of gas in the El Wastani prospect and ensuring the success of subsequent drilling operations.

In this study, the focus was on distinguishing partial gas saturation to enhance the identification of gas reservoirs, crucial for optimizing well placement and improving exploration success. By integrating various seismic inversion methods, we aimed to provide a consistent and reliable approach for confirming gas presence in the El Wastani prospect and supporting successful drilling operations.

### Post-stack inversion

Seismic inversion is a technique used to estimate subsurface properties by transforming seismic data into a quantitative rock property model. This method integrates seismic data with well data to generate acoustic impedance volumes that approximate reservoir characteristics. The inversion process involves evaluating the convolution of the reflectivity series and wavelet, as described by Eq. 1:

1 $$S(t)=r(t)*w(t)+n(t).$$

However, the type of inversion significantly influences the type of subsurface properties that can be directly estimated. Band-limited inversion, such as colored inversion, outputs band-limited impedance, which does not represent subsurface properties directly. To derive useful information, such as net pay thickness, this band-limited impedance must undergo further calibration and detuning with well data. Band-limited impedance is not a direct measure of the true elastic properties and requires an additional step to become useful for reservoir characterization. Band-limited inversion outputs band-limited impedance, which does not directly represent subsurface properties. To derive useful information, such as net pay thickness, this band-limited impedance must undergo further calibration and detuning with well data. In contrast, full-bandwidth inversion, which incorporates a low-frequency model, directly outputs true elastic properties like P-wave velocity (Vp), S-wave velocity (Vs), and density (RHOB), providing a more accurate estimate of subsurface properties. This type of inversion provides a more accurate estimate of subsurface properties without the need for additional calibration, making it highly valuable for comprehensive subsurface characterization. By incorporating low-frequency data, full-bandwidth inversion ensures the recovery of both high- and low-frequency components of the seismic signal, which enhances the overall resolution and reliability of the inversion results. This approach allows for improved reservoir characterization and aids in the accurate assessment of subsurface conditions^52,53^.

#### Inversion analysis

**Fig. 9:**
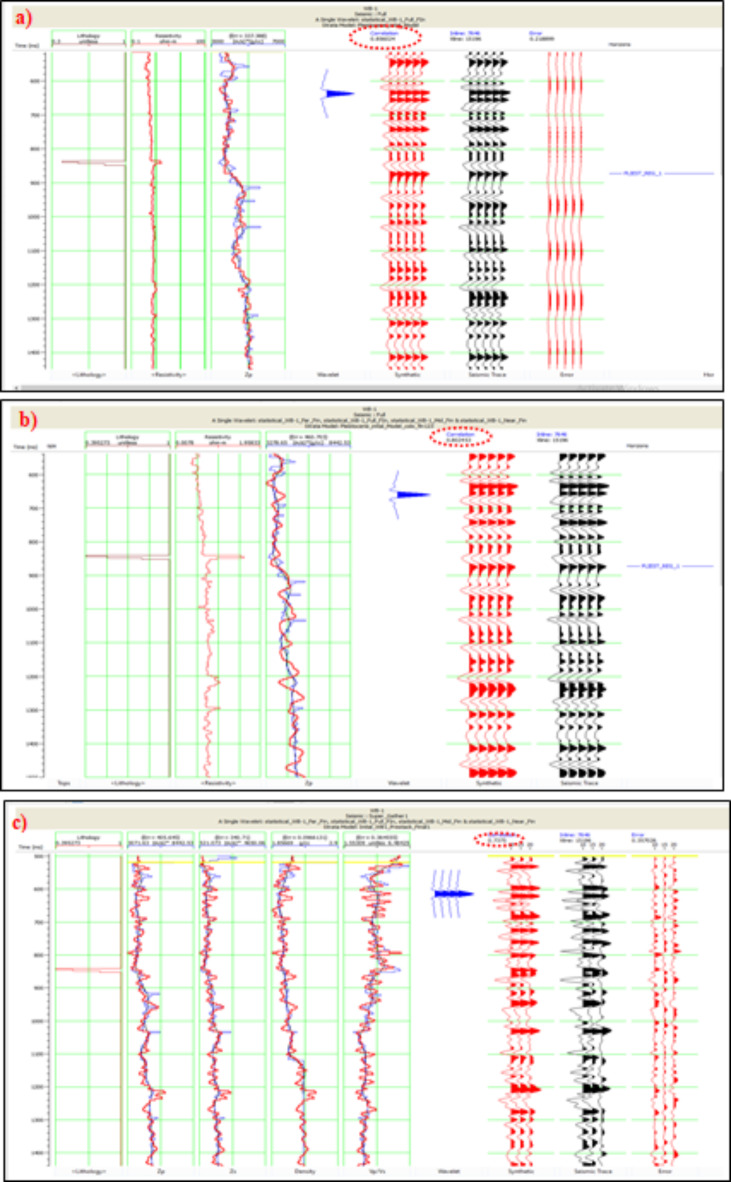
(**a**) Analysis of post-seismic inversion (model-based inversion) for the WB-1 well, focusing on the Pleistocene anomaly, with an 85% correlation. Curves are color-coded as follows: blue for the original log, black for the initial model, and red for the inverted log, using Hampson-Russel Software. (**b**) Analysis of post-seismic inversion (Sparse spike inversion) for the WB-1 well, focusing on the Pleistocene anomaly, with an 80% correlation, with the same color-coding. (**c**) Analysis of pre-seismic inversion for the WB-1 well, showing approximately 73% correlation.

The analysis conducted in this study serves as a quality control assessment by comparing seismic-inverted impedance to well-based impedance. It is essential to perform inversion analysis before seismic inversion to validate the accuracy of the results using both seismic stack and synthetic data. The analysis involves cross-correlating the initial log data (blue curve) with the inverted acoustic impedance (red curve), using the principal inverted acoustic impedance (Zp) for post-stack inversion. The process also includes determining secondary inverted acoustic impedance (Zs), density (ρ), and the ratio of primary wave velocity to shear wave velocity (Vp/Vs). For the Pleistocene section, the inversion analysis was conducted between 500 ms and 1500 ms, achieving a correlation of 73% for pre-stack inversion and 85% for post-stack inversion (Fig. 9). The black curve in the analysis represents the fit between the red and blue curves.”

### Post-stack inversion is classified into four major types

#### Colored inversion

Colored seismic inversion is a specific approach within the broader category of band-limited inversion. It allows for the quick and precise conversion of seismic data, interpolated grids, and structural properties into level characteristics^54^. Unlike traditional band-limited inversion, which focuses solely on frequency bands without background modeling, colored inversion emphasizes the calibration of the amplitude spectrum with impedance logs to effectively separate stratigraphic boundaries. This method is particularly useful for hydrocarbon exploration^55,56^. The colored inversion process can be divided into two steps: first, rotate the zero-phase seismic data by 90 degrees; second, calibrate the data’s amplitude spectrum with impedance logs.

#### Sparse spike inversion

The earth’s reflectivity is thought to consist of a series of large spikes within a background of smaller spikes. Sparse Spike Inversion posits that only significant spikes (impedance increases and decreases) occur. It combines high-resolution spikes from wells with seismic data to better resolve seismic signals by analyzing the seismic trace to identify these notable spikes^53,56^.

#### Band-limited inversion

Band-limited inversion is a specific inversion technique that targets seismic data within a defined frequency range, generally excluding the use of a low-frequency model. This approach prioritizes wavelet estimation, seismic-to-well correlation, and the statistical analysis of log-derived acoustic impedance, P-wave velocity, density, and spectral analysis of seismic data. It also necessitates the creation of an initial 3D geological model. This method bears similarities to spectral decomposition, which converts time-domain data into the frequency domain to optimize the selection of frequencies for identifying features like thin layers or gas sands^36,57–59^.

Within this framework, the initial 3D geological model acts as a structural guide, aiding the inversion process by providing spatial context, including faults, horizons, and other geological features derived from seismic interpretation and well logs. While it lacks low-frequency information, this 3D model is essential for accurately positioning geological features and ensuring consistency with established geological structures throughout the inversion process. The inversion relies exclusively on high-frequency seismic data, allowing impedance estimates to be derived from these high-frequency components directly. This technique is particularly adept at detecting fine geological features and tuning effects, such as those associated with thin layers or gas sands.

In this research, Hampson-Russell software was utilized, specifically configured to handle only high-frequency data in accordance with band-limited inversion principles. A low-frequency model was not used; instead, the initial 3D model served as a structural reference, ensuring alignment with geological features interpreted from the seismic data. This distinction between band-limited inversion and alternative methods, such as colored inversion (which improves stratigraphic resolution by calibrating amplitudes with impedance logs), underscores the emphasis on high-frequency seismic data in this study. Furthermore, a subsequent step is necessary to translate the band-limited impedance into valuable reservoir properties, highlighting the importance of thorough calibration and analysis.

#### Model based inversion

Model-based inversion is a seismic inversion technique that incorporates a low-frequency geological model to compute acoustic impedance from seismic data. This approach, often referred to as blocky inversion, combines high-frequency seismic data with a low-frequency model to refine impedance estimates. It leverages convolutional theory, where seismic traces are generated by convolving a wavelet with a reflectivity function. By integrating well logs, seismic wavelets, and a low-frequency model, model-based inversion enhances the resolution and accuracy of the acoustic impedance cube, providing a more detailed geological interpretation^27^.

### Pre-stack inversion

Pre-stack inversion differs fundamentally from post-stack inversion as it utilizes seismic angle gathers, enabling the simultaneous estimation of P-wave impedance (P-impedance), S-wave impedance (S-impedance), and density^28,60,61^. Unlike post-stack inversion, which typically estimates only P-impedance and lacks detailed information on the VP/VS ratio, pre-stack inversion provides a more comprehensive analysis by including density. This capability allows for more accurate reservoir parameter estimation and better differentiation between gas and water saturations. The integration of density measurements in pre-stack inversion enhances the overall understanding of subsurface conditions, making it particularly beneficial for the El Wastani prospect.

## Results and discussion

This study aimed to confirm the presence of gas in the Pleistocene Kanaria anomaly at the El Wastani Formation. We analyzed data from two productive wells, Wb-1 and Tersa-1 ST, which intersected the anomaly. Petrophysical analysis identified gas zones and assessed geological characteristics. Both pre-stack and post-stack inversion techniques were used to establish rock physics parameters such as density and acoustic impedance.

This study aimed to confirm the presence of gas in the Pleistocene Kanaria anomaly at the El Wastani Formation. We analyzed data from two productive wells, Wb-1 and Tersa-1 ST, which intersected the anomaly. Petrophysical analysis identified gas zones and assessed geological characteristics. Both pre-stack and post-stack inversion techniques were used to establish rock physics parameters such as density and acoustic impedance.

### Comparison of inversion techniques

The analysis highlighted the effectiveness of both inversion techniques. Traditional seismic attributes like amplitude versus offset (AVO) indicated Class III gas sand, corroborated by pre-stack inversion, which provided a more detailed view of the subsurface and more accurate identification of gas-bearing zones. Pre-stack inversion, by calculating P-wave and S-wave impedances along with density, was instrumental in selecting optimal well locations. In contrast, post-stack inversion, while useful, generally offered less resolution as it only provides acoustic impedance (Zp) or shear impedance (Zs), which may not be sufficient for comprehensive reservoir characterization. This distinction underscores the advantage of pre-stack inversion in delineating gas sands due to its direct account of impedance and density variations.

### Inversion methods comparison

Band-Limited Inversion is advantageous because it focuses on the frequency content of the seismic data, which helps in reducing the impact of noise and better matching the data’s bandwidth. In contrast, Model-Based Inversion, Sparse Spike Inversion, and Color Inversion incorporate geological assumptions and prior knowledge, making them more sensitive to geological context and potentially more reliable for detailed geological interpretations.

### Geological likelihood and uncertainty

**Fig. 10:**
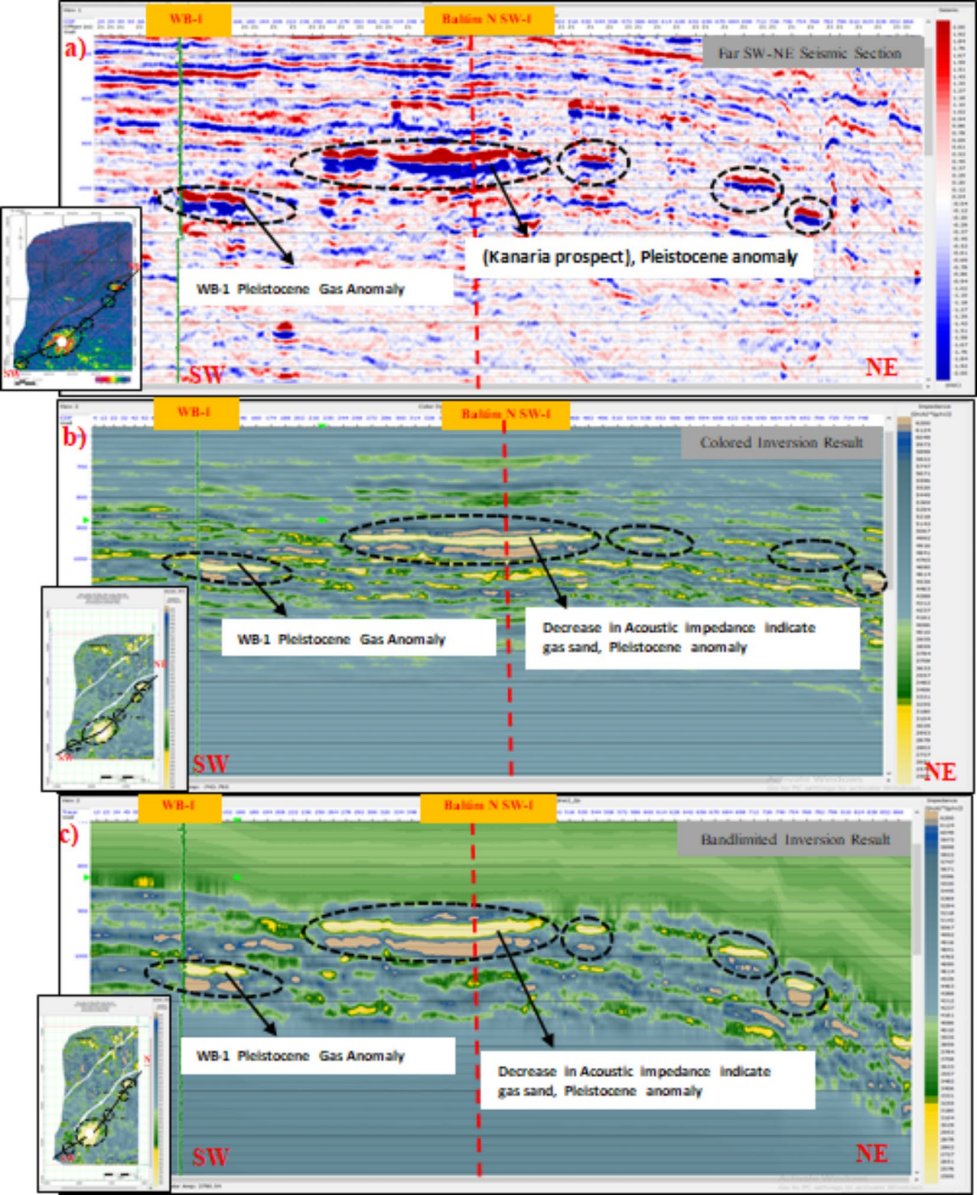
(**a**) SW-NE seismic sections through the Pleistocene anomaly (El Wastani Formation). (**a**) Full offset stack with regional amplitude map (-10, 10 ms). (**b**) Colored inversion model with time slice for Pleistocene gas sand anomaly. (**c**) Full-bandwidth inversion model for the Pleistocene gas sand anomaly, overlaid by a time slice extracted from the inversion result. All analyses were performed using Hampson-Russell Software.

**Fig. 11:**
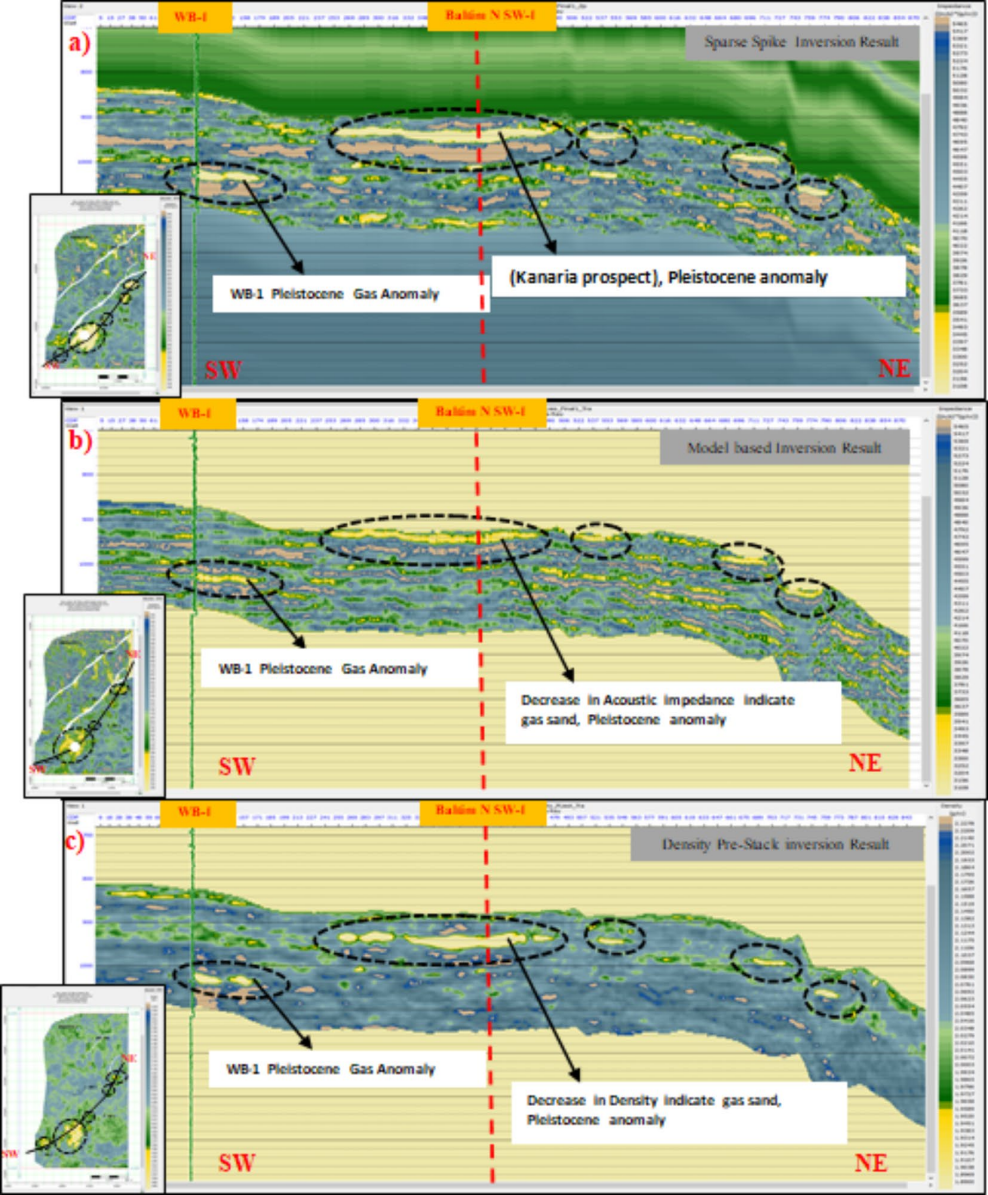
(**a**) SW-NE seismic sections through the Pleistocene anomaly (El Wastani Formation). (**a**) Sparse spike inversion model with time slice for Pleistocene gas sand anomaly. (**b**) Model-based inversion with time slice for Pleistocene gas sand anomaly. (**c**) Density pre-stack inversion model with time slice for Pleistocene gas sand anomaly. All analyses were performed using Hampson-Russell Software.

The inversion results were visually represented with low impedance values for gas sands, highlighted in yellow and pale orange on model slices (Figs. 10 and 11). Higher magnitudes in far amplitude data compared to near amplitude data supported the identification of the gas-bearing Pleistocene anomaly. Cross-plots distinctly separated gas sands from brine sands and shale. However, inherent uncertainties persist. Low saturation gas zones can mimic the seismic signatures of high saturation zones, making differentiation challenging. Additionally, geological factors such as unusually hard overlying shale can create high impedance contrasts that might be mistaken for gas sands.

### Integration of data and uncertainty mitigation

To address these uncertainties, we integrated petrophysical data from well logs with seismic inversion results. This cross-verification refined our predictions and enhanced our understanding of the reservoir characteristics. Combining AVO attributes with pre-stack inversion improved interpretation accuracy and reduced uncertainty in identifying gas-bearing zones.

### Economic considerations

Although a detailed economic analysis was beyond this study’s scope, challenges related to thinly bedded intervals with low permeability-thickness (kh) values were noted. These conditions could significantly affect gas extraction and the commercial viability of the reservoir. Further detailed reservoir characterization and economic assessment are recommended before making final drilling decisions.

## Conclusion

The primary objective of this research was to identify and confirm the presence of gas-bearing sands in the Pleistocene anomaly of the El Wastani Formation. Through seismic mapping and well-tie analysis, the target zone was refined to the Pleistocene anomaly using European polarity and zero-phase data. Seismic attribute analysis, particularly RMS Amplitude, proved effective in evaluating the Pleistocene prospects, as it demonstrated greater far-amplitude magnitudes compared to near-amplitude, indicating the presence of Class III gas sand.

Seismic inversion was employed in two forms: post-stack and pre-stack inversion. Post-stack inversion can utilize various seismic volumes, including full-offset, far-offset, or specific angle stacks like intercept and gradient volumes, depending on the study’s objectives. Each method has its advantages suited to different aspects of subsurface analysis. In this study, post-stack inversion was performed using the full-offset stack as input, providing a broader view of the subsurface by combining near, mid, and far offsets. While this approach is effective for general subsurface imaging and identifying large-scale features, it may dilute certain fluid-related features that could be more prominent in specific offset ranges. The post-stack inversion helped derive acoustic impedance, while pre-stack inversion, incorporating near, mid, and far stacks, provided a more detailed extraction of rock physics properties, including density.

Pre-stack inversion was particularly valuable in predicting rock physics parameters and confirming the presence of gas-bearing sands in the Pleistocene anomaly. The inversion’s density prediction (RHOB) matched the WB-1 well’s petrophysical evaluation, confirming low-density zones indicative of gas. This result reduced the risk of misinterpreting partial gas saturation, providing a clearer distinction between full gas and partial gas zones. The inversion revealed low-density zones in the Pleistocene interval, consistent with the density and neutron logs from the WB-1 key well, as confirmed by petrophysical evaluation and supported by figures. This correlation strongly reinforced the gas presence within the interval. Furthermore, the inversion results guided optimal well placement by highlighting zones of low acoustic impedance and high far amplitude, aligning with the high-structure contour map.

However, it is important to note that both wells used in the inversion processes may influence expectations of accuracy, as the presence of blind wells would allow for a more objective assessment of the inversion outcomes. While both post-stack and pre-stack inversions produced different volumes of rock properties and impedances, without blind wells, it remains challenging to definitively claim that one method outperforms the other in predicting rock properties away from well control. The suite of results obtained from running multiple inversion types provides valuable insights into the uncertainty surrounding predicted reservoir properties.

While post-stack inversion was useful, it has limitations due to its reliance on the full-offset stack as input and the absence of detailed logs, such as seismic and density logs from earlier wells. Pre-stack inversion also encountered challenges, particularly due to the lack of shear wave data, which, though calculable, results in less precise outputs.

## Data Availability

Data can be requested from Mr. Aly Saeed Ali El-sayed, the first author of the article; (Email: ayehia2@petrobel.org, aliyehia665@yahoo.com)

## Abbreviations

BN-3: Baltim North-3 well
BNE-1dir: Baltim North East-1 directional
EGPC: Egyptian General Petroleum Corporation
NE: North East direction
NNE: North North East direction
NW: North West direction
VSh: Shale volume
ΦT: Total porosity
ΦE: Effective porosity
Φ: Porosity (as fraction)
ρ: Density
μ: Shear modulus
Sw: Water saturation
Vp: P-wave velocity
Vs: S-wave velocity
Kframe: Bulk modulus of porous rock frame
Kmatrix: Bulk modulus of mineral matrix
Ksat: Bulk modulus of saturated rock
Kfl: Bulk modulus of pore fluid
Kclay: Bulk modulus of clay
Kqtz: Bulk modulus of quartz
ρmatrix: Density of the matrix
ρclay: Density of clay
